# Bovine Dendritic Cell Activation, T Cell Proliferation and Antibody Responses to Foot-And-Mouth Disease, Is Similar With Inactivated Virus and Virus Like Particles

**DOI:** 10.3389/fvets.2020.00594

**Published:** 2020-09-30

**Authors:** Valeria Quattrocchi, Juan Bidart, Ana Clara Mignaqui, Vanesa Ruiz, Alejandra Ferella, Cecilia Langellotti, Mariela Gammella, Sergio Ferraris, Jorge Carrillo, Andres Wigdorovitz, Yves Durocher, Sabrina Beatriz Cardillo, Bryan Charleston, Patricia Inés Zamorano

**Affiliations:** ^1^IVIT, INTA, CONICET, Buenos Aires, Argentina; ^2^IFAB, INTA, CONICET, San Carlos de Bariloche, Argentina; ^3^Centro de Ciencias Veterinarias, Universidad Maimónides, Buenos Aires, Argentina; ^4^CIA - INTA Castelar, Buenos Aires, Argentina; ^5^Human Health Therapeutics Research Center, National Research Council Canada, Montreal, QC, Canada; ^6^Biogénesis-Bagó S.A., Buenos Aires, Argentina; ^7^BBSRC National Virology Centre, The Pirbright Institute, Woking, United Kingdom

**Keywords:** FMDV, empty capsids, dendritic cells, bovine, A/ARG/2001

## Abstract

Foot-and-mouth disease (FMD) is a highly contagious disease of cloven-hoofed animals that causes severe economic losses in the livestock industry. Currently available vaccines are based on the inactivated FMD virus (FMDV). Although inactivated vaccines have been effective in controlling the disease, they have some disadvantages. Because of these disadvantages, investigations are being made to produce vaccines in low containment facilities. The use of recombinant empty capsids (also referred as Virus Like Particles, VLPs) has been reported to be a promising candidate as a subunit vaccine because it avoids the use of virus in the vaccine production and conserves the conformational epitopes of the virus. Mignaqui and collaborators have produced recombinant FMDV empty capsids from serotype A/ARG/2001 using a scalable technology in mammalian cells that elicited a protective immunity against viral challenge in a mouse model. However, further evaluation of the immune response elicited by these VLPs in cattle is required. In the present work we compare the effect that VLPs or inactivated FMDV has on bovine dendritic cells and the humoral response elicited in cattle after a single vaccination.

## Introduction

Foot-and-mouth disease (FMD) is an infectious-contagious, acute, and febrile disease of cloven-hoofed animals such as cattle, pigs, sheep, and deer, whose etiologic agent is Foot-and-Mouth Disease Virus (1, 2). The disease is endemic in many parts of the developing countries and is absent in countries from North and Central America, Australia, and Europe. FMD results in, loss of productivity and severe restrictions to international trade and pose a serious and constant threat to livestock industries, since FMD spreads rapidly giving rise to large scale outbreaks (3).

Vaccination with the inactivated virus is still the main strategy for disease control in countries where the disease is endemic (4, 5). Although inactivated vaccine has been effective in controlling the disease, it has some disadvantages. Among them, the need for expensive biosecurity facilities for vaccine production which requires constant investments in manufacturing plant up-grades and personnel qualifications together with the risk of viral escape from the production facilities or live virus contamination of the vaccines. Also, the storage and supply of the inactivated vaccine are cold-chain dependent because of the low vaccine stability at ambient temperatures and the production requires high purification process to remove the non-structural proteins from vaccine formulations to be able to discriminate vaccinated from infected cattle (6, 7). Moreover, many countries have regulatory restrictions that prohibit the production of inactivated FMD vaccines in their mainland. Because of these disadvantages, investigations are being made to produce vaccines as immunogenic as the inactivated viral vaccine in low containment facilities. FMDV recombinant empty capsids (VLPs) seem a promising alternative since they contain all the protein immunogenic sites of the virus, but lack the infectious nucleic acid and natural FMDV empty capsids have been shown to be as immunogenic as virions. Mignaqui et al. (8) have produced recombinant FMDV serotype A/ARG/2001 VLPs with high yield, using Transient Gene Expression (TGE) in mammalian cells. These empty capsids triggered a protective immune response against viral challenge comparable to the response elicited by the same amount of inactivated virus in a mouse model. A key step to move forward through the development of a novel vaccine against FMD based on VLPs is to demonstrate that the immune response elicited by these novel recombinant antigens is similar to the response to inactivated virus in the natural host.

In this regard, dendritic cells (DCs) are key players in initiating immune responses after infection or vaccination since they have unique abilities to stimulate naïve T cells and thus represents an important immune response player to study together with the immune response in the host animal.

In the present work, we compare the effect of FMDV recombinant empty capsids produced by Mignaqui and collaborators, with the response from inactivated virus on bovine DCs and the humoral response they induce in bovines, in order to gain insight about the immunogenicity of these novel recombinant particles in the natural host.

## Materials and Methods

### Experimental Design

Afferent lymph dendritic cells (ALDCs) and peripheral blood mononuclear cells (PBMCs) were obtained from the same prime vaccinated calf as described below. These cells were used to test *in vitro* VLPs immunogenicity by assessing dendritic cell activation and T cells proliferation and to compare it with the response induced by inactivated FMDV contained in the commercial vaccine. Other groups of naïve calves were vaccinated using VLPs or inactivated antigen, both formulated with commercial adjuvant, in order to compare the antibody response of both formulations.

### Harvesting ALDCs

Holando-Argentino calves were used to obtain afferent lymph dendritic cells (ALDCs) as described before (9). Briefly: 2 months before cannulation surgery, prescapular lymph nodes were removed. After that time, cannulation surgery was performed on the vessel resulting from anastomosis between afferent and efferent vessels, by inserting a cannula. Lymph was collected in a flask containing heparin and antibiotics. The flasks were replaced daily and lymph cells were concentrated by centrifugation on histopaque-1083 (Sigma) gradient. Cells were cryopreserved with fetal calf serum 10% DMSO under liquid nitrogen until use. Approximately 10–15% of the lymph cells were ALDCs characterized by flow cytometry as DEC205+/FSC^high^/CD11c+/CD8– as reported previously (9, 10). Calves were vaccinated once with the commercial vaccine regularly used in the national vaccination campaign against FMD, a few months prior to cannulation.

### Peripheral Blood Mononuclear Cells (PBMCs) Isolation

Heparinized blood was obtained by jugular vein puncture (from cannulated animal). Blood was centrifuged on Lymphoprep (Ficoll-Paque Plus 1.077 g/ml GE Healthcare) gradient. The white band containing PBMCs was washed and cryopreserved with fetal calf serum 10% DMSO under liquid nitrogen until use.

### Recombinant Empty Capsids Production

VLPs were obtained as described previously (8). Briefly, suspension-growing 293-6E cells were transiently transfected with pTT5-P12A3C plasmid, cells were harvested 48 h post transfection. After centrifugation, cell pellets were lysed by freeze and thawed cycles and analyzed for protein expression. These lysates were used as source of empty capsids and lysates from non-transfected cells were used as mock control.

### Inactivated Virus Production

FMDV serotype A/ARG/2001 was grown in BHK-21 cell cultures in Biogénesis-Bagó high biosecurity facilities. Then inactivation was carried out by Binary Ethylenimine (BEI) treatment and inactivated FMDV (iFMDV) was purified by ultrafiltration/diafiltration.

### Regulation of Co-stimulatory Molecules on ALDCs

**Figure 1:**
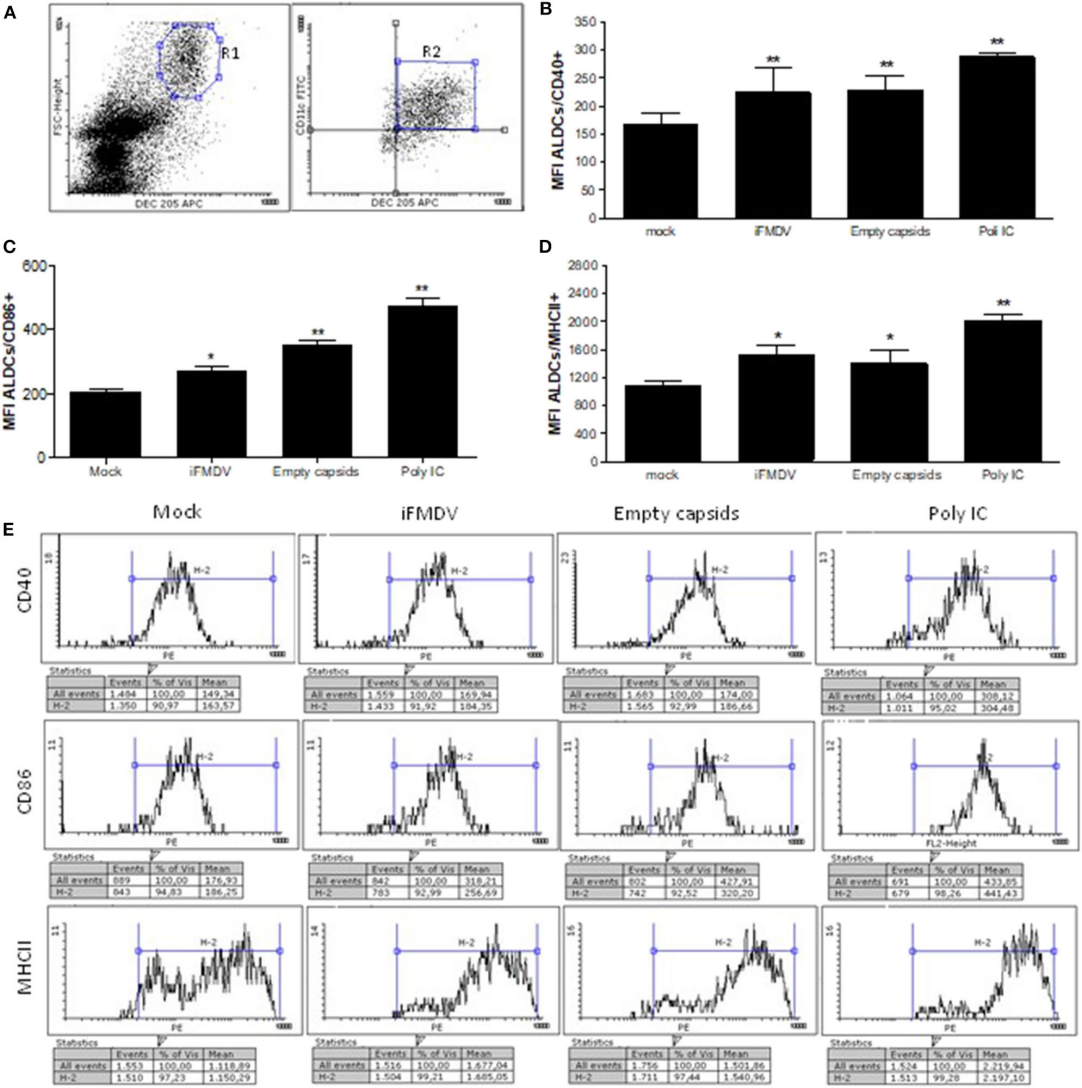
Surface expression of CD40, CD86, and MHCII co-stimulatory molecules on afferent lymph dendritic cells (ALDCs) (FSC^high^/DEC205+/CD11c+) after overnight incubation with iFMDV or empty capsids, is shown. **(A)** Gating strategy used for cytometric analysis. Co-stimulatory regulation molecules were analyzed in R2 region **(B)** Regulation of CD40 molecule **(C)** Regulation of CD86 molecule. **(D)** Regulation of MHCII molecule. Results in **(B–D)** are expressed as the mean fluorescence intensity plus Standard Deviation (SD) of four replicates in two independent assays. Mock group were lymph cells incubated with supernatant from non-transfected cells. Poly (I:C) was used as a positive control. *Significant difference *p* < 0.05, **significant difference *p* < 0.01. **(E)** One representative histogram of each group is shown.

lymph cells were incubated for 16 h with iFMDV serotype A/ARG/2001, VLPs, mock or Poly I:C as positive control, in comparable amounts (1 μg/ml) in IMDM (Gibco) cell culture medium with 10% fetal calf serum (FCS). Regulation of co-stimulatory molecules CD40, CD86, and MHCII, was measured after overnight incubation of lymph cells with antigens using an indirect surface staining performed with mouse monoclonal antibodies anti CD40, CD86, and MHCII (SEROTEC), and then anti mouse IgG-Pe conjugated (BD). Finally anti-DEC205 APC-conjugated and anti CD11c FITC-conjugated monoclonal antibodies were added. Cells were fixed with 0.2% paraformaldehyde and were acquired using FACScalibur cytometer and CellQuest software (BD). The analysis of co-stimulatory molecules expression was detected specifically in the FSC^high^/ DEC205+/CD11c+ cell population. Gating strategy is shown in Figure 1A.

### Proliferation Assessment

iFMDV or VLPs stimulated ALDCs (same procedure as used for regulation of co-stimulatory molecules), were washed with PBS and then with fresh RPMI 1640 (Gibco) 10% FCS medium, and put in contact with Carboxyfluorescein succinimidyl ester (CFSE)-charged PBMCs from the same animal. PBMCs proliferative response was evaluated according to the progressive CFSE staining reduction by flow cytometry, after 5 days incubation at 37°C in a CO_2_ incubator. Incubation of PBMCs with Concanavalin A (ConA) was used as positive control.

### Humoral Response Assessment

Seronegative calves 8–10 months old, were vaccinated by subcutaneous route with a final volume of 2 ml/dose of formulations containing 25 μg/dose of iFMDV or VLPs (*n* = 4, per group) with a water-in-oil single emulsion adjuvant included in the commercial vaccine currently used in eradication campaigns in Argentine (Biogenesis-Bagó). Calves were bleed at 15, 25, 35, and 45 days post vaccination (dpv) and humoral response were measured by liquid phase ELISA (11). Briefly: Greiner Microlon® plates were coated overnight at 4°C with 1/4,000 dilution of rabbit anti-FMDV serum in carbonate-bicarbonate buffer, pH 9.6. After washing with 0.05% Tween-20/phosphate buffered saline (PBST), plates were blocked with PBST/1% ovalbumin (blocking buffer) for 30 min at 37°C. Sera were serially diluted (first dilution 1:10 and then 1:5 dilutions) in blocking buffer and a fixed amount of iFMDV was added. After 1 h incubation at 37°C with shaking, the virus-antibody mixtures were transferred to the blocked plates, and incubated for 1 h at 37°C. A 1/1,000 dilution of guinea pig anti-FMDV serum in PBS/2% normal bovine serum/2% normal rabbit serum was added for detection, followed by 1 h incubation at 37°C. Plates were washed and peroxidase-conjugated anti-guinea pig IgG (Jackson ImmunoResearch®) serum 1/2,000 diluted in the same buffer was added, followed by 1 h incubation at 37°C. OPD/H_2_O_2_ was used as peroxidase substrate and A492 was measured in a microplate reader. Positive and negative bovine reference sera were included in each test, for validation. Antibody titers were expressed as the negative log_10_ of the highest dilution of serum that causes more than 50% inhibition of color development than in the control samples.

### Statistics

ANOVA and Bonferroni post ANOVA tests were used to compare data among groups. *P* < 0.05 was considered as an indicator of significant difference.

### Ethics Statement

Experiments involving animals were performed in accordance with protocols approved by the INTA's Ethical Committee of Animal Welfare (CICUAE Permit numbers: 06/2013).

## Results

### FMDV Empty Capsids Up-Regulates Co-stimulatory Molecules

Lymph cells were incubated with the same concentration of iFMDV or empty capsids. After staining of cells with fluorochrome-conjugated antibodies, anti DEC205, anti CD11c, and MHCII, CD40 or CD86, the analysis of co-stimulatory molecules was made on FSChigh/ DEC205+/CD11c+ cells. As shown in Figure 1, iFMDV and empty capsids significantly up-regulates co-stimulatory molecules CD40, CD86, and MHCII. As expected, Poly I: C significantly upregulated the expression of the three molecules.

### ALDCs Incubated With FMDV Empty Capsids Are Capable of Stimulating PBMCs Proliferation

**Figure 2:**
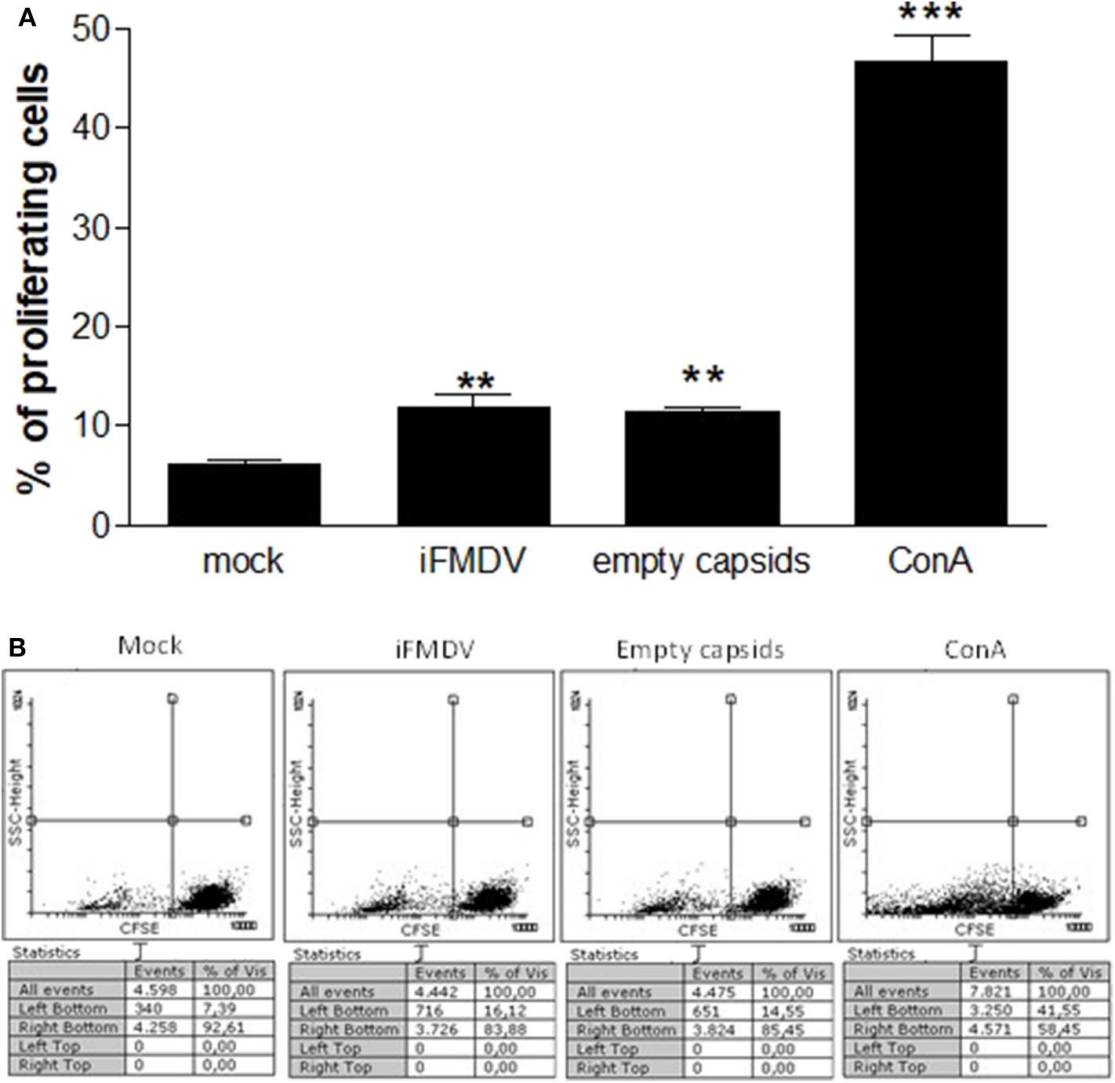
Co-cultures of lymph cells incubated with iFMDV or empty capsids, and CFSE-labeled PBMCs. **(A)** Results are expressed as percentage of proliferating PBMCs. Mock = ALDCs incubated with supernatant from non-transfected cells. ** and *** represent a significant difference (*p* < 0.01 and *p* < 0.001 respectively) regarding mock control. Mean of triplicates + SD of one representative graph of two independents assays, are shown. **(B)** A representative dot plot of CFSE lost for each group is shown.

ALDCs were incubated with the same concentration of iFMDV or empty capsids, then cells were washed repeatedly in order to eliminate any free inactivated virus or capsids not associated with the ALDCs. The ALDCs were then put in culture with CFSE-labeled PBMCs from the same animal. After 5 days incubation, an anamnestic response was detected. The percentage of proliferating T cells from a vaccinated calf was significantly increased, according to the measurement of CFSE stain loss (Figure 2). Concanavalin A was added directly on PBMCs as a positive proliferation control.

### FMDV Empty Capsids and IFMDV Are Capable of Stimulating a Similar Humoral Response in Bovines

**Figure 3:**
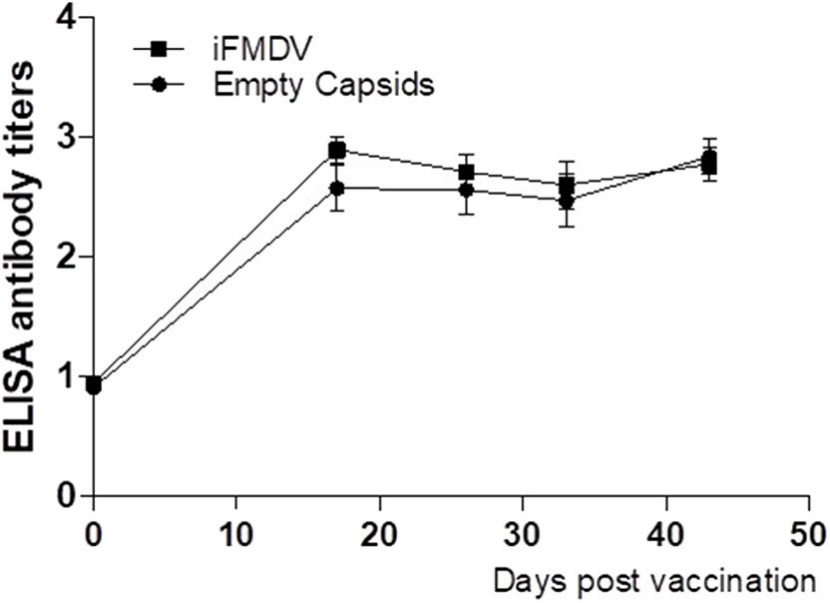
Antibodies titers against FMDV evaluated at 0, 15, 25, 35, and 45 dpv by Liquid phase ELISA in vaccinated cattle. Bovines were inoculated with inactivated FMDV (■) or empty capsids (●) formulated with commercial adjuvant. Results are expressed as mean plus SD error bars of 4 animals.

A dose of 25 μg of iFMDV or empty capsids was used according to previous reports indicating that commercial tetravalent vaccines inducing good levels of protection contain approximately this amount of inactive virus (12). When empty capsids or iFMDV were formulated with commercial adjuvant and inoculated in FMDV seronegative bovines, a specific humoral response was induced in both groups. Antibody titres (log_10_) ranged between 2.60 and 2.84 for empty capsids vaccinated group, and 2.60–2.9 for iFMDV group. In both groups, these titres of antibodies were maintained up to 45 dpv, without booster immunization (Figure 3).

According with the National Service of Health and Agricultural Quality (SENASA), these antibody titers correspond to a percentage of expected protection between 91.6 and 96.6% (13).

## Discussion

In the last years, considerable efforts have been made to develop an effective and safe vaccine against FMD, especially concerning the need to avoid the use of large quantities of live virus because of the risk and the cost of production. Since natural FMDV empty capsids have been shown to be as immunogenic as virions, several approaches have reported the use of empty capsids as an alternative vaccine. In 2013, Mignaqui and collaborators, produced FMDV recombinant empty capsids from serotype A/ARG/2001 in a mammalian expression system, and evaluated them in the mouse model that has been widely used and it has been proved to be a useful tool to predict the immune response against FMDV in bovines (14–17). Humoral responses elicited by FMDV empty capsids in mice were high and empty capsids were fully protective against viral challenge (8). Nevertheless, immune response elicited to these empty capsids has not been assessed in bovines.

This study was undertaken to evaluate the ability of FMDV recombinant empty capsids serotype A/ARG/2001, to stimulate bovine dendritic cells and consequently generate a protective humoral immune response, in order to determine if these VLPs would be a good antigen candidate for vaccine development.

DCs play a main role in the adaptive immune response development, since they are highly specialized in taking, processing and presenting antigens to naive T lymphocytes (18). *In vitro* differentiated DCs from monocytes (MoDCs) are a broadly accepted model, nevertheless, it has been reported that they functionality could be affected by the treatments used in order to differentiate them (9, 19–21). Moreover, MoDC are different cells from conventional DC (cDCs) (22) that differentiate in peripheral tissues only in inflammatory conditions, and they do not migrate in the lymph or do it weakly (23) while ALDC are mainly cDC. Charleston and collaborators (9) have developed a technique to cannulate bovine afferent lymphatic vessels, allowing collecting of large volumes of lymph containing afferent lymph dendritic cells (ALDCs). This work was carried out using ALDCs which are reported to be a more physiological alternative to *in vitro* differentiated dendritic cells, since they are not subjected to long periods of culture, enzymatic treatment or separation treatments (9, 10).

It has been reported that VLPs contain the complete repertoire of FMDV epitopes with the particulate and repetitive structure of the virus (4, 24). We investigated whether VLPs and inactivated virus would induce similar immune responses. These are important studies to understand if the inactivated RNA genome still present in the inactivated virus provided additional immune stimulation compared to empty capsids. In this sense, we demonstrate that empty capsids are capable of up-regulating co-stimulatory molecules CD40, CD86, and MHCII on the ALDCs surface in the same way as inactive virus. It is known that CD40 signaling induces changes in DCs that make them more effective antigen presenting cells, such as up-regulation of MHC class II and co-stimulatory molecules CD80 and CD86 (25, 26). Accordingly, incubation of ALDCs charged with VLPs or inactive virus, produced a proliferative response when they were incubated with CFSE- labeled PBMCs from the same animal. Taking into account that the donor calf for ALDCs and PBMCs, received one vaccination with the FMD commercial inactivated vaccine previous to the cannulation surgery and the PBMCs period of *in vitro* culture, we hypothesize that the observed proliferation correspond to memory T cells being stimulated by ALDCs. These results indicate that FMDV VLPs are processed and presented to T lymphocytes in a very similar way to iFMDV.

As expected, VLPs were able to produce a high humoral response like the iFMDV when antigens were formulated with a commercial oil adjuvant and antibody response was assessed *in vivo* in FMDV seronegative cattle. Indeed, the humoral response elicited by VLPs lasted the same time post vaccination as the one elicited by iFMDV, in the period analyzed.

Even though the animals were not challenged, the antibody titers achieved by VLPs and iFMDV are above the passmark of approval in potency test of FMD vaccines in Argentina according to statistical correlation previously reported (27).

Our results are in agreement with data from other groups, who reported that recombinant empty capsids of FMDV from different serotypes, produced in different systems, were capable of inducing humoral responses and even fully or partially protecting natural hosts against challenge (3, 28, 29).

Considering the immunogenicity of these VLPs in cattle and the fact that their production is scalable and simple, these empty capsids are a promising antigen to replace the current inactivated vaccine.

## Data Availability Statement

The raw data supporting the conclusions of this article will be made available by the authors, without undue reservation.

## Ethics Statement

The animal study was reviewed and approved by INTA's Ethical Committee of Animal Welfare (CICUAE Permit numbers: 06/2013).

## Author Contributions

VQ: experimental design, fulfillment, analysis and interpretation of *in vitro* experiments, manuscript drafts, agree to be accountable for all aspects of the work in ensuring that questions related to the accuracy or integrity of any part of the work are appropriately investigated and resolved. JB: vaccines formulation for calves, assessment and analysis of antibodies by liquid phase ELISA, and critical revision of manuscript. AM, VR, AF, YD, and AW: production, characterization, purification, and quantification of FMDV empty capsids and critical revision of manuscript. CL and MG: post-surgical cares and daily lymph collect, collaboration in flow cytometry assays, and critical revision of the manuscript. SF and JC: veterinary surgeons, control of anesthetics, cannulation surgeries, and post-surgical cares. SC: inactivated virus production and purification, purification and quantification of FMDV empty capsids used to inoculate bovines, and adjuvant provision. BC: training in cannulation surgery technique and characterization of ALDCs, critical revision of the manuscript, and approval for publication of the content. PZ: conception of the work, participation in experimental design, critical revision of the manuscript, approval for publication of the content, agree to be accountable for all aspects of the work in ensuring that questions related to the accuracy or integrity of any part of the work are appropriately investigated and resolved. All authors: contributed to the article and approved the submitted version.

## Conflict of Interest

SC was employed by the company Biogénesis Bagó. The remaining authors declare that the research was conducted in the absence of any commercial or financial relationships that could be construed as a potential conflict of interest.
